# Supplementary material for: Spontaneous ordering of identical materials into a triboelectric series
Source: Nature. 2025 Feb 19;638(8051):664–9. doi: 10.1038/s41586-024-08530-6 (PMC11839458; doi:10.1038/s41586-024-08530-6)
Supplement: Supplementary file 1 — This document contains eight Supplementary Information sections. The topics addressed are: (1) Young’s modulus measurements; (2) humidity-bias measurements; (3) time decay; (4) forcing a cycle; (5) inability to force a series with staggered contact sequence; (6) further surface-sensitive measurements; (7) roughness-bias measurements; and (8) a discussion of historical observations about mechanical (contact) history and roughness [file 41586_2024_8530_MOESM1_ESM.docx]

Supplementary Information for:

Spontaneous Ordering of Identical Materials into a Triboelectric Series

Juan Carlos Sobarzo^1^, Felix Pertl^1^, Daniel M. Balazs^1^, Tommaso Costanzo^1^, Markus Sauer^2^, Annette Foelske^2^, Markus Ostermann^3^, Christian M. Pichler^3^, Yongkang Wang^4^, Yuki Nagata^4^, Mischa Bonn^4^ and Scott Waitukaitis^1^

*^1^ Institute of Science and Technology Austria, 3400 Klosterneuburg, Austria*

*^2^Analytical Instrumentation Center, TU Wien, 1060 Vienna, Austria*

*^3^Centre of Electrochemical and Surface Technology, 2700 Wiener Neustadt, Austria*

*^4^Molecular Spectroscopy Department, Max Planck Institute for Polymer Research, Mainz 55128, Germany*

**Introduction**

This document contains Supplementary Information pertinent to the main draft, as well as the figure captions for the Extended Data. The topics addressed are: (1) Young’s modulus measurements, (2) Humidity-bias measurements, (3) Time decay, (4) Forcing a cycle, (5) Inability to force a series with staggered contact sequence, (6) Additional surface-sensitive measurements, (7) Roughness-bias measurements, and (8) a Discussion of historical observations regarding mechanical (contact) history and roughness

**Young’s modulus measurements**

We measure the Young’s modulus of an individual PDMS sample by using a materials testing device (ZwickRoell, zwickiLine 2.5 kN) to push a steel sphere of radius $R=$5 mm into the surface and measure the force as a function of indentation distance, $\delta$. Considering negligible deformation of the sphere, Hertzian contact theory predicts the following form for the force *vs.* indentation

$F= \frac{4}{3}\frac{E}{1-\nu^{2}}R^{1/2}\delta^{3/2}$, (1)

where $F$ is the measured force, $E$ is the Young’s modulus of the PDMS, and $\nu=0.5$ is its Poisson’s ratio. As $E$ is the only unknown in this equation, we obtain an accurate estimation for it by fitting our measured data to Suppl. Eq. 1. Young’s moduli for such tests with 16 samples are presented in Extended Data Figure 1. Averaging these, we find a mean and standard deviation of 4.3±0.2 MPa. Extended Data Figure 1 additionally includes data for samples after they have experienced 200 contacts. The correspondence of the Young’s modulus values before/after illustrates that the Young’s modulus does not change as a result of prior contacts. We remark that with one batch of PDMS, we observed a mild cyclic hardening, but this was not reproducible. Considering the pressure setpoint in the contact charging experiments is 45 kPa, the macroscopic strain in the CE experiments is approximately 1%. According to our own data and several other sources in the literature^1–3^, the linear elastic range for similarly prepared PDMS is up 10-20% strain. This indicates that samples are nominally within the linear elastic regime during the CE experiments. We point out however that, owing to the concentration of stresses on high-frequency features of surface roughness, the linear elastic regime might be exceed locally during contact. This could contribute in part to the observed high-frequency smoothing presented in Fig. 4h.

**Humidity**-**bias measurements**

To probe sensitivity to environmental history, we prepared 48 pristine samples with our regular procedure and stored half of them in a small chamber at $90\% \mathrm{RH}$ for a period longer than a week. The remaining half was stored in the main chamber of the experiment at $30\% \mathrm{RH}$. We then brought the high-humidity samples back to the main chamber and performed contact with these against the ‘normal’ samples. The data in Extended Data Figure 2 correspond to the charge acquired by the ‘high-humidity’ samples. As can be seen, these charged systematically negatively. Even so, we can exclude time-dependent variations in environmental conditions as a cause for the spontaneous ordering of the series due to the fact that ‘normal’ samples only ever experience the same environmental history, which is tightly regulated for the entirety of their lifetimes. Additionally, the observed humidity bias is significantly smaller than the contact bias (Fig. 2b).

**Time decay**

We produce new pristine samples (A-N) in order to investigate the time dependence of the contact bias. First, we bias sample A against sample B with 100 contacts. Then, we measure $\Delta Q_{5}$ for A against C, D, E, and so on, with defined intervals of time. This data is shown in Extended Data Figure 3, where observe a slight decrease of the bias over a period of days. However, the bias still is significantly larger than the scale of charge transfer between pristine samples (grey band in Extended Data Figure 3).

**Forcing a cycle**

In the same way that we can force the appearance of a series (Fig 3d), we can also force the appearance of a cycle. For this purpose, we produce new pristine samples E, F, G and X. We start by biasing sample E against X with 25 contacts. Then we bias sample F against sample E with 25 contacts while measuring $\Delta Q_{5}$. Then we bias sample G against sample F with 150 contacts while measuring $\Delta Q_{5}$. Finally, we measure $\Delta Q_{5}$ between G and E. The result is the cycle shown in Extended Data Figure 4.

**Inability to force a series with staggered contact sequence**

With the same samples that we used for forcing the alphabetical series (Fig. 3d), we subsequently measure $\Delta Q_{5}$ for all pair combinations again, but this time using the staggered contact sequence corresponding to the series in Fig. 1 (see Methods). As expected, the result is *not* the alphabetical series as in the main draft, nor is it even a perfect series (Extended Data Figure 5). The alphabetical series was forced by always ensuring certain samples had more contacts than others. Without this artifice, we can no longer predict the outcomes of charge transfer.

**Additional surface-sensitive measurements**

In the main text, we present data from several surface sensitive techniques in an attempt to detect differences between pristine samples and ones that have experienced 200 contacts. In all cases aside from the power spectrum of the roughness (Fig. 4h), those tests showed no significant differences. As a sanity check, we performed many more such measurements with additional sets of samples: (a) ones that have experienced several thousand contacts, and (b) ones that have been exposed for one minute to a mild oxygen plasma (Harrick Plasma cleaner PDC-002-CE, RF power 45W, duration 1 minute). The rationale behind (a) was to see if the differences in the samples could be made larger via more contacts. The rationale behind (b) was to treat samples in a way that would subtly affect their elemental, molecular, and physical properties, hence giving a benchmark for the kinds of small changes that are detectable.

Extended Data Figure 6 shows additional LEIS data for pristine, 200-contact, 2000-contact, and plasma treated samples, where the different panels correspond to different depths in the sample as achieved by ion ablation of the surface. At all depths, there are no significant differences between the pristine, 200-contact, and 2000-contact samples. We do however see large changes in the plasma-treated samples, which for shallow depths have significantly more oxygen, most likely due to the addition of OH groups. After approximately 10 atomic layers, the ratio of the O/Si in the plasma treated sample starts to approach the pristine case. Hence, even after 2000 contacts, the elemental composition of contacted samples is indistinguishable from pristine ones, and this is true for the outermost surface and several atomic layers into the bulk.

Extended Data Figure 7 probes the effect of 2000 contacts and plasma treatment on the molecular properties of sample surfaces. Panel a compares the Raman spectra, which reveals no significant differences in all cases. Panel b shows SFG spectra for the same three types of samples. In this case, the pristine and 2000 contact samples again appear identical, but differences appear in the plasma sample. These are consistent with a re-orientation and/or reduction in the methyl groups at the interface. The fact that these changes are observable in with SFG (which only probes the outermost molecular layer) but not with Raman (which probes several microns into the bulk) indicates plasma only alters a small (molecular scale) distance into the surface. This is consistent with the changes seen in the LEIS data, where alterations in the oxygen concentration become less apparent deeper into the sample.

Extended Data Figure 8 shows further results from the SEM and AFM tests. We observe that, visually, the SEM and AFM scans for 2000-contacted samples are indistinguishable from pristine samples. The plasma, on the other hand, has a visible effect, rendering the AFM data noticeably depleted of high-frequency features. When we use the AFM to calculate power spectra, as in Fig. 4h, the situation becomes clearer. The 2000-contacted sample is now even smoother at high frequencies than the 200-contacted sample, consistent with a progression in high-frequency smoothing with the number of contacts. The PSD of plasma-treated sample is significantly lower than all others, consistent with the fact that high-frequency features are visibly absent for its corresponding AFM image.

**Roughness-bias measurements**

To probe for the effect of surface morphology, and in particular motivate the plausibility for changes in roughness as a precursor to the evolution of the TE series, we prepared 48 new samples with two distinct values of roughness. To create these from the same PDMS mixture, we had to modify the usual fabrication protocol described in the Methods. We first prepared a glass Petri dish with half of its bottom inner surface roughened with hydrofluoric acid. The other half was untreated, hence extremely smooth. We cleaned the Petri dish with multiple rinses in acetone, ethanol, and Milli-Q® water. To prevent adhesion with PDMS, we dressed the dish with a monolayer of hydrophobic molecules^4^. This was achieved by first plasma cleaning the dish, and then placing it in a desiccator with a volatile drop of trimethylchlorosilane. We then let the dish sit on a shelf in laboratory conditions for more than a week. We cured PDMS in this dish and cut samples from either the rough or the smooth sides, and then attached these to sample holders in the usual way. This resulted in half of the samples having a roughness order $R_{q}\approx1$µm while the other half had roughness $R_{q}\approx1$nm. We then contacted the ‘rough’ samples against the ‘smooth’ (‘normal’) samples. The data in Extended Data Figure 9 corresponds to the charge of the rough samples. As can be seen, these charged systematically positively—hence the smoother samples charged negatively. This is qualitatively consistent with the fact that contact-biased samples (a) charge negatively and (b) are smoother (at high spatial frequencies) than uncontacted samples. Even so, the magnitude of this ‘gross’ roughness bias is less than the typical magnitude of the saturated contact bias (Fig. 2d). Bridging this gap is beyond our current understanding, but might involve considerations of the total contact area, the magnitude of local strain, *etc.*, all of which depend on the spectral properties of the roughness.

**Discussion of historical observations regarding mechanical (contact) history and roughness**

Many investigations in the literature suggest the effects we observe due to mechanical history and surface morphology are widespread, at least for polymers, yet the connection between the two has not been widely considered. For instance, when two like polymers are rubbed together asymmetrically, such that one is affected over a tiny area and the other over a large area, the former typically charges negatively to the latter^5^. This effect has been attributed to localized heating^5^, depletion of electronic trap states^6^, or generalized ‘mosaic models’^7^, but to our knowledge no have not established that this may be caused by or at least concomitant with changes to surface morphology. Other experiments have found that same-material CE for rubbing between identical solution-cast (*i.e.* smooth) polymers is small in magnitude and erratic, whereas after mechanical lapping (*i.e.* roughening) it becomes large and systematic^8^. In practical settings, *e.g.* for triboelectric energy harvesting, samples are sometimes ‘conditioned’ until their charge exchange ‘behaves’ by application of many (*e.g.* hundreds or even thousands) pre-contacts^9^. In at least one situation where polarity reversals have been observed with different materials, it has been due to prolonged frictional interaction. The authors of that study concluded this was due to material transfer, although they simultaneously observed changes in surface morphology^10^. Experiments incriminating surface morphology date at least as early as 1867, when Sir William Snow Harris reported that pristine (smooth) glass charged with a different sign relative to other materials than glass roughened by abrasion^11^. Here an interesting distinction must be pointed out, which is that the smooth glass tends to charge positively*,* not negatively. (We reiterate that work in our own lab suggests the behavior of oxides, *e.g.* glass, may be quite different than polymers.) In more recent work with polymers whose roughness was changed by solution precipitation or casting/dissolution of embedded sugar granules, the trend that ‘smooth polymers charge negatively’ was shown to be widespread^12,13^. Further careful experiments to test the connection between contact history, surface morphology, and charge exchange are required to definitively connect our findings to these earlier results, and establish whether the ‘self-ordering’ we observe is widespread.

**References**

1. Moučka, R., Sedlačík, M., Osička, J. & Pata, V. Mechanical properties of bulk Sylgard 184 and its extension with silicone oil. *Sci Rep* **11**, 19090 (2021).

2. Johnston, I. D., McCluskey, D. K., Tan, C. K. L. & Tracey, M. C. Mechanical characterization of bulk Sylgard 184 for microfluidics and microengineering. *J. Micromech. Microeng.* **24**, 035017 (2014).

3. Glover, J. D., McLaughlin, C. E., McFarland, M. K. & Pham, J. T. Extracting uncrosslinked material from low modulus sylgard 184 and the effect on mechanical properties. *Journal of Polymer Science* **58**, 343–351 (2020).

4. Baytekin, H. T. *et al.* The Mosaic of Surface Charge in Contact Electrification. *Science* **333**, 308–312 (2011).

5. Shaw, P. E. Electrical separation between identical solid surfaces. *Proc. Phys. Soc.* **39**, 449–452 (1926).

6. Lowell, J. & Truscott, W. S. Triboelectrification of identical insulators. II. Theory and further experiments. *Journal of Physics D: Applied Physics* **19**, 1281–1298 (1986).

7. Grosjean, G. & Waitukaitis, S. Asymmetries in triboelectric charging: Generalizing mosaic models to different-material samples and sliding contacts. *Phys. Rev. Materials* **7**, 065601 (2023).

8. Lowell, J. & Truscott, W. S. Triboelectrification of identical insulators. I. An experimental investigation. *J. Phys. D: Appl. Phys.* **19**, 1273–1280 (1986).

9. Ģērmane, L. *et al.* Physical and Chemical Surface Modification of Recycled Polystyrene Films for Improved Triboelectric Properties. *Energy Tech* **12**, 2400762 (2024).

10. Baytekin, H. T., Baytekin, B., Incorvati, J. T. & Grzybowski, B. A. Material Transfer and Polarity Reversal in Contact Charging. *Angew Chem Int Ed* **51**, 4843–4847 (2012).

11. Harris, W. S. *A Treatise on Frictional Electricity, in Theory and Practice*. (Virtue, London, 1867).

12. Verners, O. *et al.* Smooth polymers charge negatively: Controlling contact electrification polarity in polymers. *Nano Energy* **104**, 107914 (2022).

13. Šutka, A. *et al.* Contact electrification between identical polymers as the basis for triboelectric/flexoelectric materials. *Phys. Chem. Chem. Phys.* **22**, 13299–13305 (2020).
